# Ocular Morpho-Functional Evaluation in ATTRv Pre-Symptomatic Carriers: A Case Series

**DOI:** 10.3390/diagnostics13030359

**Published:** 2023-01-18

**Authors:** Martina Maceroni, Benedetto Falsini, Marco Luigetti, Angela Romano, Valeria Guglielmino, Romina Fasciani, Giorgio Placidi, Elena D’Agostino, Paola Sasso, Stanislao Rizzo, Angelo Maria Minnella

**Affiliations:** 1 Institute of Ophthalmology, Università Cattolica del Sacro Cuore, 00135 Rome, Italy; 2 Fondazione Policlinico Universitario A. Gemelli-IRCCS, 00135 Rome, Italy; 3 Institute of Neurology, Università Cattolica del Sacro Cuore, 00135 Rome, Italy

**Keywords:** transthyretin amyloidosis (ATTRv), ATTRv pre-symptomatic carriers, optical coherence tomography (OCT), OCT-angiography, electroretinogram (ERG), in vivo corneal confocal microscopy (IVCM), ocular biomarkers, personalized medicine

## Abstract

The present study aimed to investigate ocular findings in hereditary transthyretin amyloidosis (ATTRv) pre-symptomatic carriers. Fourteen ATTRv pre-symptomatic carriers, who are patients with positive genetic testing but without signs or symptoms of the disease, were retrospectively evaluated. Retinal morphology was assessed using optical coherence tomography (OCT) and OCT-angiography. Retinal function was evaluated using cone b-wave and photopic negative response (PhNR). Pupillometry and in vivo corneal confocal microscopy (IVCM) were performed. ATTRv pre-symptomatic carriers presented a significantly reduced central macular thickness (CMT) (*p* = 0.01) and outer nuclear layer (ONL) thickness (*p* = 0.01) in comparison to normal controls. No differences were found when analyzing sub-foveal choroidal thickness, retinal nerve fiber layer and ganglion cell complex. In comparison to healthy controls, pre-symptomatic carriers presented an attenuated superficial retinal vascular network and a significantly augmented PhNR amplitude (*p* = 0.01). However, PhNR implicit times, B-wave amplitude and B-wave peak time did not show significant differences in comparison to controls. No differences were found for pupillometric values. All the examined eyes presented alterations in the IVCM. Preclinical ocular structural and functional abnormalities can be found in ATTRv pre-symptomatic carriers. Thus, an extensive ophthalmological evaluation should be included at the baseline visit and during follow-up. Considering the availability of new drugs potentially able to prevent or delay disease progression, the identification of new disease biomarkers appears to be particularly promising.

## 1. Introduction

Hereditary transthyretin amyloidosis (ATTRv) is an adult-onset, autosomal-dominant, multisystemic disease caused by mutations in the gene encoding transthyretin (TTR). TTR is a plasma protein that functions as a carrier for thyroxine (T4) and retinol (vitamin A) [1].

ATTRv amyloidosis is spread globally, with endemic foci in Portugal, Sweden, Japan, Brazil, Maiorca, and Cyprus. The Italian national prevalence is 4.33/million, with higher values in southern Italy [2].

The main organs involved in ATTRv are peripheral nerves and the heart, resulting in sensory–motor neuropathy, autonomic dysfunction and cardiomyopathy. However, 10% of patients present with ocular involvement [3].

The main signs of ocular amyloidosis include vitreous opacities, chronic open-angle glaucoma (COAG), abnormal conjunctival vessels (ACVs), sicca keratoconjunctivitis (SKC), loss of corneal sensitivity and neurotrophic corneal ulcers, lens anterior capsule opacities, retinal vascular changes, pupillary light-near dissociation, irregular pupils and optic neuropathy [3]. The ocular manifestation affecting the anterior compartment of the eye (e.g., ACVs) is due to a circulating mutated protein produced by the liver. However, plasma TTR cannot cross the blood–retina barrier [4]. Thus, the manifestation affecting the posterior compartment of the eye is mainly due to local production of amyloid fibrils, especially by the retinal pigment epithelium [5]. The progression of ocular involvement after liver transplant confirms that the ocular alterations are largely related to a local production of amyloid deposits [6].

In the last few years, new drugs potentially able to prevent or delay disease progression have become available [7,8]. In this perspective, the identification of reliable disease biomarkers appears to be particularly promising. The early identification of disease onset in ATTRv pre-symptomatic carriers could allow prompt treatment as soon as minor, but clinically meaningful, disease signs are detected [9,10,11,12].

The aim of the present study was to investigate ocular findings in ATTRv pre-symptomatic carriers using optical coherence tomography (OCT), OCT-angiography (OCT-A), electrophysiology, pupillometry and in vivo corneal confocal microscopy (IVCM) in order to find novel potential ocular biomarkers of the disease.

## 2. Materials and Methods

The study was approved by the Ethics Committee/Institutional Review Board of the Catholic University (Prot. ID 4108). This research adhered to the tenets of the Declaration of Helsinki and informed consent was obtained from all patients. All the clinical, imaging, and electrophysiological data reported in this study were analyzed retrospectively. Recruitment was performed from January 2021 to September 2022 according to a collaboration protocol with the Department of Neurology of Università Cattolica del Sacro Cuore, Fondazione Policlinico Universitario Agostino Gemelli.

### 2.1. Subjects

All the subjects included were selected from a larger cohort of patients with a confirmed diagnosis of ATTRv. All the “ATTRv pre-symptomatic carriers” were included in the study. Patients were defined as pre-symptomatic carriers in the presence of an established diagnosis of ATTRv confirmed by a positive genetic testing without signs or symptoms of the disease.

Exclusion criteria were as follows: the comorbidity of diabetes, atherosclerotic vasculopathy, glaucoma, or any other macular or retinal disorders, optical media opacity precluding reliable retinal functional exams, and inability of the patients to maintain visual fixation.

### 2.2. Neurological Evaluation and Pupillometry

Global neurological evaluation and nerve conduction studies, after the genetic characterization of the pathogenetic variant, were performed for each patient.

Quantitative automated pupillary light reflex (PLR) was measured in all subjects with NPi-200 (developed by NeurOptics, Inc., Irvine, CA, USA). Baseline pupil diameter (BPD), minimum pupil diameter (MPD), reflex latency (RL), constriction velocity (CV), maximum constriction velocity (MCV), dilation velocity (DV), constriction index (CI), which is the percentage of change, calculated as (BPD−MPD)/BPD and expressed as a percentage (%), and neuro-pupillary index (NPi), which is a composite parameter integrating RL, CV, and DV, were calculated.

### 2.3. Ophthalmological Evaluation

All the enrolled patients underwent a full ophthalmologic examination, including best corrected visual acuity (BCVA) and intraocular pressure (IOP) measurements, as well as anterior segment slit lamp biomicroscopy and indirect fundus ophthalmoscopy. Color fundus photos were taken with Eidon (Centervue, Freemont, CA, USA).

#### 2.3.1. Optical Coherence Tomography (OCT) and OCT-Angiography (OCT-A)

SD-OCT was performed using Zeiss Cirrus 5000-HD-OCT Angioplex, sw version 10.0, (CarlZeiss, Meditec, Inc., Dublin, CA, USA). A high-definition 5 Line Raster, a macular map (6 × 6 mm Macular Cube 512 × 128) and optic nerve head map for retinal nerve fiber layer (Rnfl) evaluation were acquired. OCT-A imaging was performed using a 6 × 6 mm volume scan pattern centered on the fovea.

OCT qualitative assessment was performed by two independent masked investigators (A.M.M. and M.M.) who evaluated macular scans in search of vitreous opacities, vitreo-retinal interface and outer retina alterations. Central macular thickness (CMT) was automatically measured using macular cube scans. Subfoveal choroidal thickness (SFCT) was manually measured on horizontal OCT B-scans, with calipers measuring the distance from the posterior edge of the RPE to the choroid–sclera junction. Outer nuclear layer (ONL) thickness was manually measured at 5 points from the posterior edge of the outer plexiform layer (OPL) to the external limiting membrane (ELM) at the fovea, and at 1.500-micron and 3000-micron intervals temporal and nasal to the fovea. An average thickness for ONL was calculated from the 5 values obtained. Ganglion cell complex (GCC) average thickness was automatically measured using ganglion cell analysis from the macular cube. GCC includes the ganglion cell layer and inner plexiform layer (IPL). An image of the superficial capillary plexus (SCP) and deep capillary plexus (DCP) was generated using automated layer segmentation, corrected by manual readjustments of the segmentation lines. SCP 6 × 6 vessel density (VD) was expressed as a percentage derived from the ratio of the total vessel area (all white pixels, defined as pixels with a ratio value between 0.7 and 1.0) to the total area of the analyzed region (size of the image in pixels). Vessel perfusion (VP) was defined as the total area of perfused retinal microvasculature per unit area in a region of measurement. FAZ perimeter was calculated as the length of the contour based on pixel-to-pixel distance in a scale and was expressed in millimeters. The area of FAZ was measured by counting the total number of pixels within FAZ in a scale multiplying the dimension of a pixel and expressed in square millimeters. FAZ circularity was also registered.

Twenty-six eyes of thirteen healthy patients were evaluated as controls for OCT and OCT-A measurements.

#### 2.3.2. Electroretinogram Assessment

Ganzfeld cone-mediated (light adapted) electroretinograms (ERGs) were recorded in all patients. For each patient, ERGs were recorded according to a published protocol employed to isolate and analyze the PhNR from the single-flash cone-mediated responses [13,14,15]. The amplitude and peak time of the cone b-wave and the PhNR were measured in each recording session. The instrument used was Retimax, (CSO Company, Florence, Italy). A group of 40 healthy eyes with no signs of any ocular disease served as controls

#### 2.3.3. In Vivo Corneal Confocal Microscopy (IVCM)

IVCM was performed with HRT laser Rostock cornea module (Heidelberg, Germany). A qualitative evaluation was performed by one expert investigator (R.F.) who analyzed the subepithelial nervous plexus (extension and density), the nerve segmentation and/or fragmentation (increased beads) and the branch density.

### 2.4. Statistical Analysis

We analyzed both right and left eyes. For statistical reasons, we considered in the analysis only the results from the right eyes. Values were expressed as frequencies (%), mean ± standard deviation (SD) or median [interquartile range] as appropriate. Results were analyzed by parametric tests (*t*-test and analysis of variance) assuming normal distribution. A conservative *p*-value = or < 0.01 was considered to be statistically significant.

## 3. Results

Among all the patients with a genetic diagnosis of ATTRv followed up with at the Neurology Department, 14 patients (28 eyes) met all the requirements of the inclusion/exclusion criteria. The mean (range) age of patients was 54 (38–73) years and 5 (36%) patients were men, whereas 9 (64%) patients were women. TTR gene sequencing revealed Val30Met pathogenic variant in five patients, Phe64Leu in seven patients, andAla120Ser and Glu89Gln in one patient each. The main demographic and genetic data of the study sample are summarized in Table 1.

### 3.1. Neurological Examination and Nerve Conduction Studies

Clinical examination was unremarkable in each subject. Nerve conduction studies were normal in all cases excluding peripheral neuropathy.

### 3.2. Pupillometry

No differences were found for pupillometric values between ATTRv pre-symptomatic carriers and healthy controls [16]. Results from automated pupillometry are reported in Table 2.

### 3.3. Anterior Segment

Best corrected visual acuity (BCVA) was substantially preserved in all subjects, with a mean value of 84 ETDRS letters (range 57–90). Three eyes presented a decreased BCVA because of cataracts in two cases and keratoconus with corneal apex opacification in one case. Intraocular pressure (IOP) was within normal limits in all subjects, with a mean IOP of 16 mmHg. None of the examined patients had a diagnosis of glaucoma. Slit lamp biomicroscopy of the anterior segment was unremarkable in all the examined eyes, with the exception of the abovementioned cases of cataract and keratoconus. Two of the examined eyes were pseudophakic. Data are reported in Table 1.

### 3.4. Posterior Segment

Fundus examination did not reveal vitreous opacities or vascular abnormalities of the retinic vascular arcades in all the examined patients. Three eyes showed RPE dystrophic alteration in macular region, whereas one eye showed vitreoretinal interface abnormalities (Table 1).

### 3.5. OCT and OCT-A Assessment

**Figure 1:**
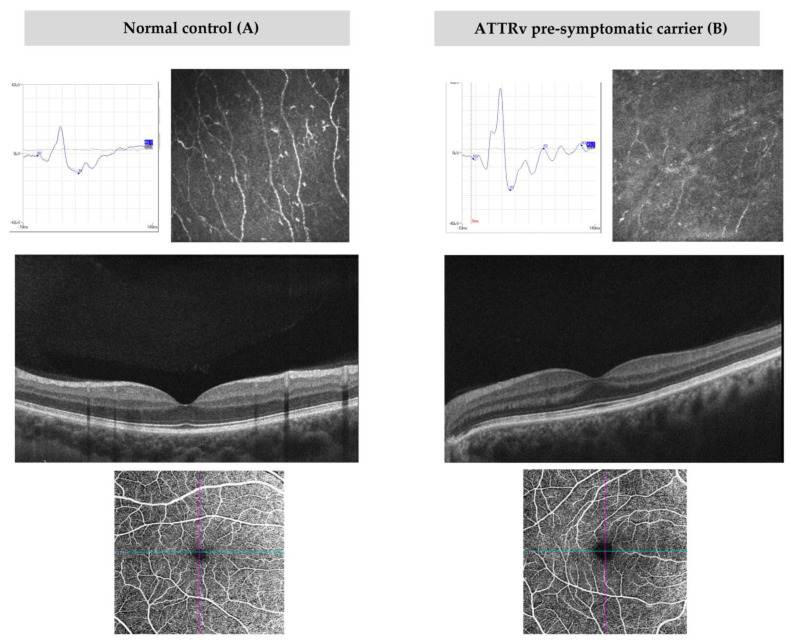
Top to bottom: photopic electroretinogram (ERG), in vivo corneal confocal microscopy (IVCM), OCT B scan and OCT-A images of the superficial capillary plexus (SCP) of normal controls (**A**) and pre-symptomatic ATTRv carriers (**B**). (**A**) shows normal PhNR values (10.52 mV), as well as normal nervous corneal plexus. OCT B scan reveals preserved morphology and reflectivity of all retinal layers, with a mean ONL thickness of 79 microns. OCT-A enface images of the SCP reveal a vessel density of 19.8 mm/mm^2^. (**B**) shows supernormal PhNR (18.52 mV), with an altered corneal nervous plexus. No alterations in retinal morphology and reflectivity are observed on OCT scan. Mean ONL thickness is 61 microns and VD SCP is 17 mm/mm^2^.

Regarding retinal OCT assessment, 26 normal control eyes of 13 healthy patients (5 males, 7 females) were evaluated for comparison. OCT findings are reported in detail in Table 3. In the ATTRv pre-symptomatic carriers, qualitative assessment of B-scan OCT images did not reveal vitreal abnormalities; vitreo-retinal interface alteration consisting of a macular cellophane were found in three eyes (11%). Notably, four eyes showed pachychoroid-spectrum OCT abnormalities, with RP epitheliopathy in all cases associated with subretinal fluid in the macular region in one eye. In ATTRv pre-symptomatic carriers, CMT was significantly decreased (251.35 ± 18.05 μm vs. 266.15 ± 11.61 μm, *p* = 0.01), while SFCT did not differ in comparison to healthy controls (270.85 ± 68.77 μm vs. 270.38 ± 36.75 μm, *p* = 0.9). In ATTRv pre-symptomatic carriers, ONL thickness was significantly reduced compared to normal controls (67.5 ± 5.98 μm vs. 79.87 ± 5.5 μm, *p* = 0.01). Rnfl showed a mean value of 94 ± 8.7 μm in ATTRv pre-symptomatic carriers, not differing from normal controls. None of the examined eyes presented focal defects in the optic nerve head map. GCC was similar between the two examined groups (83.92 ± 5.09 μm in pre-symptomatic carriers vs. 80.92 ± 5.4 μm in healthy controls, *p* = 0.1). Although the results were not statistically significant, pre-symptomatic carriers presented an attenuated superficial retinal vascular network in comparison to healthy controls: a reduced VD (17.5 ± 0.7 vs. 18.86 ± 0.8 mm/mm^2^, *p* = 0.05), a reduced PD (42.77 ± 5.5 vs. 45.7 ± 1.7%, *p* = 0.08), larger FAZ area (0.30 ± 0.1 vs. 0.23 ± 0.07 mm^2^, *p* = 0.4), and a larger FAZ perimeter (2.24 ± 0.4 vs. 2.09 ± 0.5 mm, *p* =0.4). Ophthalmological measurements are reported in detail for each patient in Table 3. Figure 1 shows OCT and OCT-A scans of a pre-symptomatic carrier in comparison to a normal control.

### 3.6. Electrophysiology

Pre-symptomatic carriers presented a mean photopic ERG B wave amplitude of 28.83 μV (SD 10.36), with an implicit time of 33.9 ms (SD 1.8). No significant differences were found in comparison to normal controls. However, PhNR amplitude was significantly augmented in pre-symptomatic carriers in comparison to controls (12.58 μV, SD 3.45 vs. 7.82 μV, SD 2.28, *p* = 0.01). PhNR implicit time did not show significant differences in comparison to the healthy group (48.48 μV, SD 5.3 vs. 49.58 μV, SD 2.64; *p* = 0.5, not significant). Notably, ONL thickness showed a positive correlation with photopic ERG B wave amplitude (*p* = 0.002).

Electrophysiological data are reported in Table 2. Figure 1 shows electrophysiological measures of a pre-symptomatic carrier in comparison to a normal control.

### 3.7. In Vivo Corneal Confocal Microscopy (IVCM)

Sixteen eyes of eight patients were analyzed using IVCM. All the examined eyes presented a rarefied subepithelial nervous plexus (for extension and density), nerve segmentation and/or fragmentation (increased beads) and a reduced branch density. One patient presented endotheliosis in both eyes. Details on IVCM are summarized in Table 4, and IVCM images are represented in Figure 1.

## 4. Discussion

In the present study, we identified preclinical structural and functional ocular abnormalities in ATTRv pre-symptomatic carriers. ATTRv pre-symptomatic carriers presented a significant reduction in CMT and ONL thickness and an attenuated superficial retinal vascular network. Electrophysiological parameters showed significantly augmented PhNR values, and IVCM was altered in all the examined eyes. The identification of ocular alterations in pre-symptomatic carriers without any clinical sign of systemic involvement can be plausible considering the frequent dissociation between systemic and ocular symptoms. Indeed, there is evidence that ocular manifestations do not run parallel to the entity of systemic symptoms, and this is due to the eye being capable of its own TTR production. Hara et al. followed up for a mean time of 7 years with a number of ATTR Val30Met amyloidosis patients and assessed ocular involvement after liver transplantation [17]. Liepnieks et al. provided further evidence that local synthesis of variant TTR by the RPE is responsible for vitreous amyloid formation, not TTR from hepatic production [6].

Recent studies have revealed the presence of subclinical structural and functional ocular abnormalities in ATTRv patients. In a cohort of ATTRv-affected patients with an apparently normal ocular examination, CMT and SFCT were slightly reduced, as well as ONL thickness, which was significantly reduced (*p* = 0.002) in comparison to controls (72.57 ± 8 μm vs. 79.5 ± 6.05 μm) [18]. Mixed rod–cone and cone ERG b-wave amplitudes were reduced, as well as PhNR amplitude [18]. Similarly to ATTRv patients, ATTRv pre-symptomatic carriers presented a significant reduction in CMT and ONL thickness in comparison to healthy controls. As postulated by Minnella et al., the loss of ONL could be a consequence of a reduced retinoid supply—given that TTR is a carrier for thyroxine (T4) and retinol—and a consequent reduced number of photoreceptors [18].

Concerning OCT-A findings, the sole analysis of the superficial capillary plexus—without the deep plexus—is a limitation of the study. However, the rarefaction of the superficial plexus found in pre-symptomatic carriers resembles that found by Marques et al. in a group of ATTRv patients [19]. They found that scalloped irises in ATTRv eyes are associated with a more advanced subclinical retinal angiopathy than eyes without scalloped irises [19], confirming that OCT-A may identify vascular alterations serving as potential disease biomarkers.

Amyloid deposition in the retinal and choroidal vessels has long been reported [20]. Retinal microvascular changes in ATTRv patients could be explained by considering the micro-occlusive damage derived from amyloid deposition. Although further studies would be needed to elucidate the characteristics of all the capillary plexa in a larger cohort of patients, OCT-A could be a promising non-invasive technique to identify early signs of retinal vascular involvement in ATTRv pre-symptomatic carriers.

Interestingly, we found a significant increase in PhNR amplitude in pre-symptomatic carriers. Several studies have reported supernormal ERG values in CRVO eyes with mild retinal ischemia [20]. The supernormal ERG may be caused by changes in the electrical activity of retinal cells through an ischemia-induced increase in anti-VEGF levels. Considering that retinal neurons do not express anti-VEGF receptors, supernormal ERGs may be secondary to extravasation and activation of the kallikrein-kinin system [21] or to Nitrix Oxide [22] production, both induced by VEGF.

Considering the research evidence, we speculate that the supernormal PhNR in pre-symptomatic carriers is likely to be related to chronic ischemic inflammatory damage due to microvascular alterations in the internal retina.

IVCM is a useful tool for detecting early nerve damage in vivo. In ATTRv patients, a correlation between corneal nerve damage and the severity of both sensorimotor and autonomic neuropathies has been noted [23]. Thus, corneal nerve damage detected by IVCM can be considered as a disease biomarker. A recent study conducted on five ATTRv pre-symptomatic carriers found that corneal sub-basal nerve density was low in all of the examined patients during the entire 3 years of follow-up [24]. Our results support the evidence of an altered subepithelial nervous plexus in ATTRv pre-symptomatic carriers, confirming that IVCM is a sensitive technique for identifying systemic neuropathy even when other instrumental examinations are found to be negative. Subclinical ocular abnormalities detected by IVCM in *TTR* mutation carriers could be early expression of small fiber neuropathy (SFN). Actually, IVCM has already proved to be able to detect and quantify small-fiber involvement in several peripheral neuropathies, such as diabetes, Fabry disease, and idiopathic small-fiber neuropathy (iSFN), early [25]. Analogously, this rapid, noninvasive technique may be used as a surrogate early marker of SFN in ATTRv [23], since subclinical ocular abnormalities in IVCM may sometimes even precede the loss of intraepidermal nerve fibers in skin biopsy [24,25].

The small sample size is a limitation of the study. However, considering the rarity of the condition and the lack of literature data on pre-symptomatic carriers, the sample examined could be relevant. Further studies with larger sample sizes would be needed to elucidate the ophthalmological findings in this particular population.

## 5. Conclusions

In conclusion, the results of the present study indicate that preclinical ocular structural and functional abnormalities can be found in ATTRv pre-symptomatic carriers. The identification of novel disease biomarkers appears to be particularly useful, considering that nowadays patients may benefit from treatments able to prevent or delay disease progression. Extensive ophthalmological evaluation should be included in the baseline visit and during follow-up in ATTRv pre-symptomatic carriers.

## Figures and Tables

**Table 1:** Demographic, clinical and ophthalmological data.

Case	Gender	Age	Pathogenetic Variant	BCVA (ETDRS Letters, n)	IOP	Anterior Segment	Vitreous Opacities	Ocular Fundus
1 *RE* *LE*	F	68	Val30Met	85 77	16 12	Cataract, Cataract	No No	U U
2 *RE* *LE*	M	42	Val30Met	85 83	14 15	U U	No No	U U
3 *RE* *LE*	F	45	Val30Met	85 85	18 17	U U	No No	U U
4 *RE* *LE*	M	49	Phe64Leu	86 86	19 15	U U	No No	RPE dystrophy RPE dystrophy
5 *RE* *LE*	F	43	Phe64Leu	85 85	14 16	U U	No No	U U
6 *RE* *LE*	F	70	Phe64Leu	83 71	14 12	DALK for KC, KC	No No	U U
7 *RE* *LE*	F	73	Phe64Leu	85 85	13 13	U U	No No	U U
8 *RE* *LE*	M	44	Glu89Gln	90 90	19 16	U U	No No	U U
9 *RE* *LE*	F	59	Val30Met	85 83	15 16	U U	No No	RP epitheliopathy
10 *RE* *LE*	F	72	Ala120Ser	84 84	20 20	Pseudofachic Pseudofachic	No No	U Macular cellophane
11 *RE* *LE*	F	38	Phe64Leu	87 87	19 19	U U	No No	U U
12 *RE* *LE*	M	49	Phe64Leu	85 85	18 18	U U	No No	U U
13 *RE* *LE*	M	41	Phe64Leu	90 90	20 20	U U	No No	U U
14 *RE* *LE*	F	73	Val30Met	57 85	21 20	Cataract, Cataract	No No	U U

BCVA, best corrected visual acuity; F, female; IOP, intraocular pressure; LE, left eye; M, male; RE, right eye; U, unremarkable.

**Table 2:** Electrophysiological and pupillometric findings.

Case	Photopic ERG		PhNR		Pupillometry							
	B Wave Amplitude	B Wave Peak Time	Amplitude	Peak Time	NPI	BDP	MPD	CI	CV	MCV	RL	DV
1 *RE* *LE*	31.44 18.05	33.98 33.11	11.66 9	48.93 41.43	4.7 4.7	3.44 3.53	2.25 2.28	35 35	2.80 3.18	4.27 4.74	0.23 0.20	Na Na
2 *RE* *LE*	31.86 20.34	33.69 33.98	12.69 11.86	43.07 50.1	4.1 4.2	4.22 3.63	2.93 2.74	29 25	2.06 1.87	3.73 2.74	0.20 0.20	Na 1.19
3 *RE* *LE*	10.45 19.81	34.28 33.69	7.79 10.77	50.1 49.22	3.7 3.5	5 4.57	2.47 3.36	31 26	2.43 2.80	4.27 4.04	0.23 0.23	1.57 1.18
4 *RE* *LE*	30.57 22.51	33.11 32.81	8.63 9.4	42.28 41.02	4.5 4.6	4.11 4.01	2.61 2.51	36 37	3.50 3.46	4.72 4.82	0.20 0.20	1.59 1.50
5 *RE* *LE*	38.14 32.56	33.11 33.12	11.83 9.55	50.98 49.51	4.6 4.6	4.62 4.49	2.74 2.64	41 41	2.60 3.14	4.42 4.45	0.23 0.20	1.11 1.29
6 *RE* *LE*	28.91 31.97	33.40 33.13	14.12 9.16	48.34 49.51	4.8 4.5	3.33 3.73	2.04 2.56	39 31	2.17 2.24	3.55 3.01	0.27 0.27	0.92 Na
7 *RE* *LE*	25.41 25.66	33.69 33.40	14.85 9.77	46 44.82	4.5 4.5	3.77 3.96	2.55 2.59	32 35	2.38 2.26	3.48 3.94	0.23 0.23	0.51 1.21
8 *RE* *LE*	24.26 26.98	32.81 34.11	8.83 10.03	49.22 42.19	4.6 4.7	4.30 4.30	2.56 2.49	40 42	2.64 3.14	3.75 4.44	0.23 0.17	1.31 0.96
9 *RE* *LE*	20.18 14.70	33.40 33.40	10.65 9.78	54.49 46.58	4.4 4.6	4.01 4.01	2.70 2.48	33 38	2.49 2.50	3.67 4.09	0.23 0.23	1.36 1.00
10 *RE* *LE*	27.20 30.80	33.69 33.68	13.29 12.23	49.22 48.93	4.6 4.8	3.37 2.83	2.33 1.84	31 35	2.44 1.87	3.40 3.02	0.23 0.20	0.73 0.95
11 *RE* *LE*	43.33 37.62	32.81 33.11	17.88 15.72	42.77 42.48	4.6 4.4	4.98 4.37	2.77 2.74	44 37	3.29 2.92	5.33 4.63	0.17 0.2	1.44 1.44
12 *RE* *LE*	34.55 46.20	33.40 32.81	16.37 19.07	46.58 46.88	4.3 4.3	5.57 5.44	3.31 3.28	41 40	3.38 3.62	5.61 5.42	0.23 0.23	1.55 1.45
13 *RE* *LE*	46.01 34.87	33.11 33.11	18.52 19.39	44.24 45.12	4 4.3	5.57 5.53	3.55 3.39	36 39	3.17 3.66	4.54 5.49	0.2 0.2	1.18 1.84
14 *RE* *LE*	11.36 11.38	40.14 39.55	9.11 8.47	62.4 62.7	4.4 4.2	3.48 4.04	2.86 2.55	29 27	2.53 2.09	3.36 3.29	0.17 0.23	1.05 1.12

BPD, baseline pupil diameter; CI, constriction Index; CV, constriction velocity; DV, dilation velocity; LE, left eye; MCV, maximum constriction velocity; MPD, minimum pupil diameter; NPI, neuro-pupillary index; RE, right eye; RL, reflex latency.

**Table 3:** OCT and OCT-A assessment.

Case	OCT						OCT-A			
	CMT	SFCT	ONL	GCC	RNFL	Qualitative Alterations	VD SCP (mm/mm^2^)	PD SCP (%)	FAZ AREA (mm^2^)	FAZ PERIMETER (mm)
1 *RE* *LE*	245 250	267 296	77 71	80 74	95 87	No No	18 18.8	44.1 46.3	0.18 0.19	1.64 1.8
2 *RE* *LE*	251 252	391 327	72 73	88 90	87 92	No no	19.1 19	46.1 45.9	0.39 0.33	2.64 2.43
3 *RE* *LE*	213 224	302 343	57 61	77 80	91 93	No No	17.8 18.9	44.4 47	0.49 0.41	2.81 2.6
4 *RE* *LE*	289 294	390 332	72 70	88 89	98 97	RPE dystrophy RPE dystrophy	18.8 18.4	46.6 45.8	0.14 0.14	1.6 1.55
5 *RE* *LE*	253 245	243 296	64 66	82 81	91 93	No No	18.7 18.5	44.8 44.6	0.21 0.22	1.86 1.84
6 *RE* *LE*	252 244	249 267	60 66	77 80	82 75	No No	11.9 Media opacities	28.4 Media opacities	0.42 Media opacities	2.76 Media opacities
7 *RE* *LE*	252 237	267 250	67 69	80 80	94 88	Cellophane Cellophane	14.4 16.8	35.9 41.4	0.18 0.33	1.78 2.22
8 *RE* *LE*	269 267	272 249	67 68	82 83	92 88	No No	19.3 18.7	47.1 45.8	0.18 0.19	1.63 1.76
9 *RE* *LE*	269 263	272 278	70 74	79 79	97 99	RPE dystrophy No	18.2 17.8	45.3 44.3	0.19 0.22	1.77 2.2
10 *RE* *LE*	260 259	320 278	64 63	88 87	101 95	No cellophane	18.1 18.3	45.5 45.6	0.23 0.24	2.1 2.11
11 *RE* *LE*	231 230	280 249	79 79	90 90	113 110	No No	18 19.1	44.9 47	0.44 0.44	2.77 2.64
12 *RE* *LE*	251 255	254 261	72 75	92 93	100 96	No No	19.2 19.2	47.5 46.6	0.3 0.24	2.13 2
13 *RE* *LE*	239 244	260 284	74 72	89 89	98 97	No No	18.1 17	44.3 41.2	0.36 0.29	2.51 2.24
14 *RE* *LE*	245 233	101 163	50 56	83 83	77 83	No No	15 13.6	37 32.5	0.36 0.47	2.4 2.83

CMT, central macular thickness; FAZ, foveal avascular zone; GCC, ganglion cell complex; LE, left eye; ONL, outer nuclear layer; PD, perfusion density; RE, right eye; RNFL, retinal nerve fiber layer; SCP, superficial capillary plexus; SFCT, subfoveal choroidal thickness; VD, vessel density.

**Table 4:** IVCM results.

Patients	Eye	IVCM			
		Rarefied Subepithelial NP (Extension and Density)	Nerve Segmentation and/or Fragmentation	Reduced Branch Density	Other Alterations
#1	RE	na	na	na	
	LE	na	na	na	
#2	RE	na	na	na	
	LE	na	na	na	
#3	RE	na	na	na	
	LE	na	na	na	
#4	RE	na	na	na	
	LE	na	na	na	
#5	RE	yes	yes	yes	
	LE	yes	yes	yes	
#6	RE	yes	yes	yes	endotheliosis
	LE	yes	yes	yes	endotheliosis
#7	RE	yes	yes	yes	
	LE	yes	yes	yes	
#8	RE	yes	yes	yes	
	LE	yes	yes	yes	
#9	RE	yes	na	na	
	LE	yes	na	na	
#10	RE	yes	yes	yes	
	LE	yes	yes	yes	
#11	RE	yes	yes	yes	
	LE	yes	yes	yes	
#12	RE	no	yes	yes	
	LE	no	yes	yes	
#13	RE	na	na	na	
	LE	na	na	na	
#14	RE	yes	yes	yes	
	LE	yes	yes	yes	

LE, left eye; na, not available; RE, right eye.

## Data Availability

Data are available from authors.
